# Differential Diagnosis and Management of Incomplete Locked-In Syndrome after Traumatic Brain Injury

**DOI:** 10.1155/2017/6167052

**Published:** 2017-06-14

**Authors:** Lauren Surdyke, Jennifer Fernandez, Hannah Foster, Pamela Spigel

**Affiliations:** Brooks Rehabilitation Hospital, 3599 University Blvd S, Jacksonville, FL 32216, USA

## Abstract

Locked-in syndrome (LIS) is a rare diagnosis in which patients present with quadriplegia, lower cranial nerve paralysis, and mutism. It is clinically difficult to differentiate from other similarly presenting diagnoses with no standard approach for assessing such poorly responsive patients. The purpose of this case is to highlight the clinical differential diagnosis process and outcomes of a patient with LIS during acute inpatient rehabilitation. A 32-year-old female was admitted following traumatic brain injury. She presented with quadriplegia and mutism but was awake and aroused based on eye gaze communication. The rehabilitation team was able to diagnose incomplete LIS based on knowledge of neuroanatomy and clinical reasoning. Establishing this diagnosis allowed for an individualized treatment plan that focused on communication, coping, family training, and discharge planning. The patient was ultimately able to discharge home with a single caregiver, improving her quality of life. Continued evidence highlights the benefits of intensive comprehensive therapy for those with acquired brain injury such as LIS, but access is still limited for those with a seemingly poor prognosis. Access to a multidisciplinary, specialized team provides opportunity for continued assessment and individualized treatment as the patient attains more medical stability, improving long-term management.

## 1. Introduction

It has been estimated that approximately 10 million people across the world are affected by traumatic brain injury (TBI) annually with nearly half of those individuals remaining moderately to severely disabled one year out from injury [1]. Due to the extensive care for these patients, their total burden of care costs across the lifespan has been estimated at $200 million per year when considering acute management through long-term placement [1]. Rates of misdiagnosis in patients with a disorder of consciousness (DOC) from TBI have been reported to be as high as 43% [2–5]. This rate of misdiagnosis is highest when performed by nonspecialized physicians and rehabilitation teams [5, 6]. Persons with locked-in syndrome (LIS), akinetic mutism (AM), and spinal cord injury (SCI) can also have very similar presentation making differential diagnosis complex. One of these differentials, LIS, is a rare outcome of cerebral damage that is both debilitating and complex to diagnose. Traumatic LIS only accounts for 10% of etiologies, with the most frequent cause being interruption of the motor pathways in the ventral pons by basilar artery occlusion [7]. Other reported causes include but are not limited to ALS, tumors or abscesses, and postoperative complications [7].

LIS was originally introduced by Plum and Posner in 1966 as a condition associated with lesion of the ventral pons, disrupting the corticospinal and corticobulbar pathways without involvement of the cortex [8]. In 1979, Bauer et al. introduced the notion that instead of one typical presentation of the syndrome, three varieties exist including the classical variety, incomplete variety, and total variety [9]. The classical variety manifests as quadriplegia, lower cranial nerve paralysis, and mutism with preservation of vertical gaze and, most notably, intact consciousness indicated by abilities to communicate via eye movements. The incomplete variety is similar in presentation to the classical variety; however, the patient presents with additional voluntary movements that vary on a case-by-case basis. The total variety describes a patient with no voluntary movement and closed eyes [9]. Despite the lack of apparent awareness in these patients, indications of conscious mental activity have been shown through use of technology such as EEG, which can correlate brain activity in relation to stimuli [10].

Much of the recent literature supports a need for standardized diagnostic procedures to confirm LIS; however, this is often reliant on imaging which may not show pathological changes even when a clinical picture of LIS is present [11, 12]. Several obstacles exist in correlating clinical presentations to anatomical pathology, including but not limited to the varied underlying patient characteristics (age, baseline cognitive status and orientation, previous neurological disorders, and comorbidities), as well as the diversity in size and nature of the lesion itself [13]. Oftentimes in acute care, patients are too sedated and are seen for such brief periods of time that does not allow for thorough clinical assessment.

Over the past 10 years, a growing body of literature has investigated highly dependent patients such as those with a DOC or LIS and how they may benefit from inpatient rehabilitation. Increased reports suggest that highly dependent individuals after TBI can benefit from comprehensive inpatient rehabilitation despite an initial seemingly poor prognosis and that specialized care units improve outcomes [1, 13, 14].

However, due to limited funding and the strength of research to support the need for this comprehensive level of care, many patients are not given such an opportunity. As a result, days or months often go by before an accurate diagnosis of LIS is made. León-Carrión et al. reported that the diagnosis of LIS is not usually made until approximately two months after onset [7]. This complexity increases in trauma patients where there may be multiple injures leading to greater increases in time until an accurate diagnosis [15, 16].

Clinicians can use their knowledge of neuroanatomy and expected clinical presentations to isolate a suspected lesion location and thus aid in early diagnostics when imaging is not readily available. The purpose of this paper is to describe the clinical differential diagnosis process that occurred in the inpatient rehabilitation setting for a patient presenting with quadriplegia and mutism following traumatic etiology. A secondary purpose of this paper is to highlight how access to this level of care and an accurate diagnosis impacted outcomes for this patient.

## 2. Case Description

A 32-year-old female was admitted to a specialized inpatient rehabilitation program utilizing a comprehensive rehab team focused on assessing patients with severe traumatic brain injury with the goal of providing accurate diagnosis, family training, and intensive therapy to promote best outcomes. The team included a physician, neuropsychologist, physical therapist, cognitive therapist, speech therapist, occupational therapist, and nurse. This multidisciplinary team collaborated towards the final diagnosis described here and together established a personalized plan of care and discharge recommendations.

Acute care records showed patient had undergone CT of the brain revealing right frontal parenchymal contusion and a diffuse area of subarachnoid blood. No midline shift, herniation, or mass effect was identified. Other acute comorbidities included right subcondylar mandible fracture requiring her jaw to be wired shut, further complicating assessment during her inpatient stay. No reports regarding MRI or angiography were received from the acute care setting which may have expedited diagnostics prior to admission to inpatient rehabilitation.

Initial team evaluation in the specialized program focuses on identifying and differentiating purposeful and generalized responses to stimuli using a combination of testing components derived from the Western, CRS-R, and Glasgow [16]. Observations of purposeful activity during initial evaluation were not observed due to lack of any spontaneous movement of the patient; however, the patient appeared generally awake based on observation of open eyes and spontaneous visual tracking. Ocular bobbing, which is characterized by a fast down beat of the eyes and slow return to baseline, was observed in addition to a distinct sustained and direction-changing nystagmus [17]. The functional independence measure (FIM) was used to capture the patient's disability and how much assistance the patient required to perform activities of daily living. Refer to Table 1 for further examination findings and Table 2 for FIM scoring.

Based on the patient's wakefulness and consistent meaningful and purposeful interactions with the environment through eye gaze communication, a disorder of consciousness was ruled out. Further, understanding of the anatomy of the described reflexes and presentation guided the team towards isolation of a ventral pons lesion location and the suspected diagnosis of LIS, with need for further work-up to confirm.

## 3. Differential Considerations

Based on the team evaluation, differential diagnosis aimed to include diagnoses that may match the patient presentation of acute onset of quadriplegia and mutism following trauma. The working list of diagnoses included LIS, DOC, AM, and an upper cervical SCI. Since LIS was the primary suspected diagnosis, the goal of further assessment was to rule out all other suspected diagnoses in order to confirm by exclusion. See Table 3 for a schematic of the overall differential diagnosis process.

### 3.1. Disorders of Consciousness

There are three primary classifications for DOC including coma, unresponsive wakefulness, and minimally conscious state. Consciousness is defined by being both alert and aware. Alertness depends on normal functioning of the reticular formation, thalamus, and cortex while awareness requires higher ordering processing that integrates both sensory and motor information [17]. In a DOC, some degree of impairment exists within these structures; see Table 4 for the classifications of DOC.

In the presented case of traumatic etiology, the diagnostic process was complicated by frontal lobe brain injury confirmed by CT from acute care stay. Inappropriate or absent responses could be due to language impairments, initiation impairments, or other associated impairments more likely related to the brain injury as opposed to locked-in syndrome. Based on the patient's demonstration of arousal and consistent means of communicating a level of orientation, the diagnosis of a DOC was ruled out.

### 3.2. Upper Cervical Spinal Cord Injury

An upper cervical SCI was included due to the presentation of quadriplegia. All key muscles defined by the International Standards for Neurologic Classifications of Spinal Cord Injury Association (ISNCSI) in the standard motor exam were scored zero out of five bilaterally, leading to the notion that the injury must be above the level C5 at which the motor exam begins [18]. Sensory testing was completed, using a modified method due to communication impairments. The patient was instructed to open her eyes when light touch was detected, and this was used to test all key sensory points. Light touch was detected at all key points using this method, but examination was unable to determine if the sensation was altered to any degree with this method. Additionally, sharp dull differentiation was deferred due to communication impairments and time limitations.

With an upper cervical SCI, there is also reasonable expectation for demonstration of labored breathing or need for mechanical ventilation support with injuries above C5 [19]. Our patient demonstrated quiet respiration with no abnormal patterns of inhalation. Additionally, both facial expression (controlled by cranial nerve VII in the brainstem) and head/neck control were impaired in this individual, indicating a supraspinal lesion and reducing the likelihood that the patient presentation was due to SCI.

### 3.3. Akinetic Mutism

AM is a condition characterized by diminished neurologic drive with a decrease in nearly all motor functions including facial expression, gestures, and speech output but with some degree of alertness and intact spontaneous visual tracking [20]. In this condition, individuals typically maintain normal muscle tone and reflexes; however, due to the decreased spontaneous movement a perceived paralysis could allow it to be mistaken as LIS. A study of AM following stroke revealed that eight patients with AM identified the frontal lobe as the most frequent location of lesion [20]. The frontal lobe plays an important role in the initiation of behavior and speech, and its dysfunction produces lack of spontaneity and reduced motor output. Based on the role of the frontal lobe in initiation, protective extension and equilibrium reactions would remain intact, even if delayed due to the nature of these reactions being initiated automatically and involuntarily in the brainstem [21].

In this case, these reactions were persistently impaired throughout the course of the patient's stay in addition to a lack of any withdrawal to pain. In AM, the patient should demonstrate some evidence for motoric abilities through self-initiated tasks. For example, patients with AM may be observed performing automatic motor tasks such as swatting a fly. The telephone effect has also been described in patients with AM in which the patient spontaneously answers a ringing phone with a verbal “hello” [22]. This patient did not reveal any of these characteristics of AM. In addition, the patient demonstrated initiation and the ability to follow some motor commands through specific visual activation and eye opening/closing without any delay. Over the course of care, the patient also began to demonstrate some recovery of more distal voluntary movements (trace toe and finger movements, bilateral head turning, and head lifting to midline) with the ability to initiate movement on command once movement potentials were established. Based on the presented rationale, the diagnosis of AM was ruled out.

### 3.4. Locked-In Syndrome

Clinical reasoning allowed for exclusion of above working diagnoses, supporting the likelihood that the patient presentation was due to LIS. She presented with the classical symptoms of quadriplegia, mutism, intact consciousness, and ability to communicate via eye gaze movements. Reading comprehension was determined to be intact later in her stay, further supporting the diagnosis of LIS where patients typically remain cognitively intact [23]. She also demonstrated the ability to sequence three step commands via eye gaze movements and was oriented to situational and personal questions with 100% accuracy by week five of her inpatient stay.

Knowledge of anatomy and correlation to presentation further strengthened the diagnosis of LIS by helping isolate where the suspected lesion was. The presentation of motor versus sensory impairments indicated a more likely ventral lesion [21]. The vestibular ocular reflex, which was intact, uses dorsally located connections with cranial nerves III and VI, again indicating a more ventral lesion. To further isolate a ventral lesion, a positive Babinski sign supported corticospinal tract damage [21]. Based on ISNCSI testing, the lesion was likely above C5. Absence of facial expression, which is controlled by cranial nerve VII in the brainstem, further indicates a more cephalic lesion. The pupillary light reflex was intact. This reflex descends to the pretectal area in the midbrain before reaching the oculomotor nucleus and achieving its motor output [10]. An intact pupillary light reflex indicated the lesion was likely below the midbrain. By using the above rational based on patient presentation, the lesion location was isolated to the ventral pons further confirming the diagnosis of LIS. The patient was classified under the incomplete variety due to her recovery of additional voluntary movements, which included active movement of toes, head turning and maintaining midline, and facial expression [9].

## 4. Outcomes

The highly skilled rehabilitation team was able to confirm diagnosis through clinical assessment at week five after injury, with imaging affirming a small pontine injury at week six. The patient was cared for initially in an acute care hospital with limited therapy for four weeks and no confirmed diagnosis, prior to her five-week inpatient rehabilitation admission. With a five-week stay in inpatient rehabilitation came the time that allowed for accurate and timely assessment, recognizing that the diagnosis of LIS is not usually made until two months after onset [7]. Imaging was performed early on in the patient's course of care; however, the initial CT showed no lesion associated with the ventral pons. This CT was performed without contrast and arguably too early to show the corresponding lesion associated with LIS [10]. MRI, which may have been more sensitive in detecting the lesion, was not performed until week six after injury with no reports as to why this was not performed in the acute care hospital.

Establishing the diagnosis of LIS allowed for treatment approaches and goals to shift in order to maximize functional patient participation and family training. Aggressive mobilization demonstrated limited motor recovery and decreased expectation of such with the diagnosis of LIS warranted treatment focused on improving communication to more efficiently identify and address the patient's needs. Augmentative and alternative communication was trialed via the Tobii; however, the patient was not able to functionally use this device due to difficulties associated with calibration from her resting nystagmus. Mobility training became compensatory with focus on use of a head switch power wheelchair and family training to safely assist in transfers.

Upon discharge, the patient was able to use a head controlled device to alert caregivers when she required assistance, reducing her required level of supervision. She was able to direct her care with the ability sequence multistep commands, reducing the required level of education and experience of future caregivers. She was more efficient with her altered means of communication, allowing her to interact and participate in life more. With improved communication, cognition, and postural control, the patient was also able to engage in initial phases of learning how to operate a head control power chair. Though the patient did not achieve independence with wheelchair mobility, she continued to show potential for improved functional use of a power chair with continued reinforcement of steering skills and safe obstacle negotiation. Upon discharge, she was able to navigate straight paths and wide turns; however, due to inconsistent performance and distance modifiers, she required total assistance based on the FIM.

**Figure 1:**
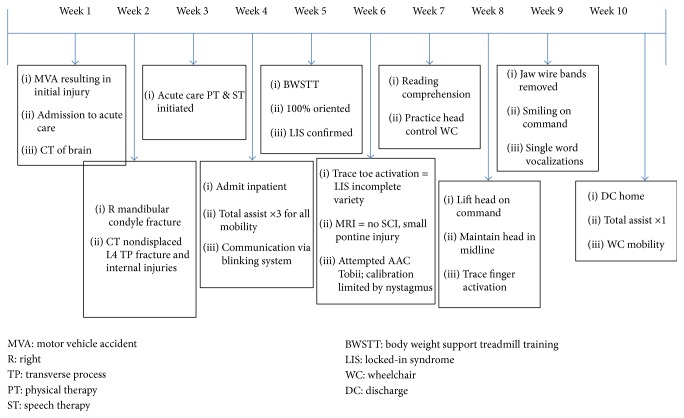
Patient course of care timeline.

Despite these meaningful functional changes, the patient's FIM scores did not adequately highlight what was achieved through rehabilitation (Table 2). She required total assistance for all self-care and mobility items with minimal but meaningful communication and cognitive progressions. Refer to Figure 1 for a review of this patient's stay, highlighting timing and outcomes.

## 5. Discussion

Access to inpatient rehabilitation in this case facilitated an early and accurate diagnosis, which streamlined the patient's individualized plan of care. Extensive family training, increased patient mobility through wheelchair propulsion, and improved communication allowed this patient to return home with family ten weeks after injury instead of requiring institutional care. The patient was further able to make decisions and direct her care, increasing her current and future autonomy. This is of great significance as these patients are often placed in long-term care facilities and at the same time are living longer [24]. Considering lifelong costs has become more important due to the reported longer life expectancy in those with LIS, understanding that up to 83% will live ten years after onset [11, 25]. Current research has demonstrated that by simplifying care needs and providing family training, inpatient rehabilitation can reduce long-term costs and in this case it did [26, 27]. Longer life expectancy additionally brings to consideration quality of life (QOL), with research to support that physical disability alone does not predict a lesser QOL [11, 25]. This patient achieved improved autonomy as stated above and was able to return home, both of which are positively associated with greater quality of life in those with LIS.

As displayed above, outcomes in this case were positively impacted by access to a comprehensive rehab program. Clinicians with expertise in treating patients with disorders of consciousness allowed for accurate assessment and diagnosis which guided individualized treatment of this particular patient. It took a multidisciplinary team effort to ensure carryover of therapeutic interventions and to reduce the likelihood of secondary complications. Imaging was performed early on in the patient's course of care; however, the initial CT showed no lesion associated with the ventral pons. This CT was performed without contrast and arguably too early to show the corresponding lesion associated with LIS. Kotchoubey and Lotze reported 22 patients with severe occlusive defect of the basilar artery in which CT did not show any pathological changes during the acute stage, reporting that hypodensity typically cannot be seen until 2 weeks after the infarct [10]. MRI, which is reported to be the most sensitive method in diagnosis of structural disorders in LIS, was not performed until week six, shortly after the patient's admission to inpatient rehabilitation. It has been reported that MRI can reveal a distinct lesion that was not visualized on CT and is particularly important for cases of nonvascular etiology, as in this case of traumatic etiology [10]. The MRI cleared the patient for SCI damage and showed a small pontine injury. Even still, in some cases in which the patient presents with clinical signs of LIS, MRI and CT scans may show no pathological changes, warranting the need for the adjunct clinical based assessment to guide treatment [28, 29].

Since reimbursement is directly tied to FIM gains, those with such a severe brain injury are not often given the opportunity to participate in inpatient rehabilitation and undergo this level of assessment. The FIM has floor effects in highly dependent individuals, which was particularly evident in this case warranting the need for more sensitive measures [13]. Research supports that an early and intensive multidisciplinary treatment plan for patients with LIS, begun within one month of onset, improved health status and decreased the chances for mortality [24] Even still, access to an inpatient rehabilitation team is limited and when provided the opportunity, pressure exists for early discharge leading to higher chances of institutionalization [13, 26]. Literature continues to support that lifelong costs are lowest following TBI with supervised home placement after rehabilitation [1]. It is then our responsibility as health care professionals to advocate for these individuals to maximize access and ultimate outcomes following diagnosis of LIS.

## 6. Conclusion

Continued research is needed to better define what low level patients can gain from inpatient rehabilitation and health care providers must recognize the importance of accurate diagnosis for plan of care development, noting that an individualized and team approach are critical in the management of poorly responsive patients. Limitations in health care coverage and funding for high level studies will make this challenging, with high rates of misdiagnosis further confounding research. New models of assessment and care for these patients must be established to maximize their opportunity for making functional and meaningful progress to lessen their burden of care and public cost impacts. There is an identified need for a more standardized approach and more sensitive means of tracking functional progress in these patients to obtain reimbursement. Though case reports alone will not be strong enough to facilitate development of standards of care, well researched case reports can continue to build the body of literature upon which future research can grow.

## Figures and Tables

**Table 1:** Examination findings.

Expressive communication	No verbalizations or facial gestures, able to establish communication via blinking, 75% accuracy regarding orientation

Visual tracking	All directions, nystagmus and ocular bobbing noted

Arousal/attention	Awake, alert, focused on examiner throughout

Auditory response	No motor response to commands except ocular

Object manipulation	No motor response or grasp reflex noted

Motor response	No head control, righting reactions, or protective extension. No withdrawal to pain.

Reflexes	VOR and pupillary light intact

**Table 2:** FIM scoring.

FIM scores	Evaluation	Discharge
Self-care	8/56	8/56
Mobility	5/35	5/35
Communication and cognition	5/35	17/35
Total	18/126	30/126

**Table 3:** Differential diagnosis: acute onset of quadriplegia and mutism.

Diagnoses considered		Key finding to rule out	
Disorder of consciousness		Assessment revealing patient wakefulness and ability to communicate via eye gaze	*✕*
	↓		
Upper cervical spinal cord injury		Observation of normal, quiet respiration and impairment of supraspinal muscles	*✕*
	↓		
Akinetic mutism		Lack of automatic protective extension/equilibrium reactions and no withdrawal to pain	*✕*
	↓		
Locked-in syndrome		Primary suspected diagnosis by exclusion of other likely diagnoses	✓

**Table 4:** Primary classifications for disorders of consciousness.

Specific disorder of consciousness	Defining features
Coma	Unconscious and unaware with disruption of the reticular activating system of the brainstem.
Unresponsive wakefulness	Partially conscious and no awareness, with preservation of brainstem structures.
Minimally conscious state	Limited but clear evidence for awareness of self/environment with inconsistent but reproducible goal-directed behaviors. Brainstem structures intact.
